# Avoidable Diseases

**Published:** 1893-11

**Authors:** C. B. Newton

**Affiliations:** Stafford Springs, Conn.


### HALL'S JOURNAL OF HEALTH

HEALTH—THE POOR MAN'S RICHES, THE RICH MAN'S BLISS.

VOL. XL. NOVEMBER, 1893. NO. 11.

### AVOIDABLE DISEASES.

The Board of Health are doing a great work. They point out to the people the diseases which can be avoided through their various health reports. In these publications an interest is awakened. The people are learning that most diseases are avoidable. This is a fact that the reading and thinking class now recognize. One of the best illustrations of this we have had this past season.

An exhaustive scientific study of cholera has been made, enabling our government to arrest the disease. We have learned its characteristics as we would those of a common enemy.

Our consuls at foreign ports have been very vigilant as well as sanitary officers at our ports of entry. The condition of the coast cities were looked to and organic decaying matter removed that it might not become a culture medium. Close, crowded and ill-ventilated tenement houses remain, but are no cause for alarm if the germ of cholera is not planted there, neither is the water supply of a city liable to be contaminated as the source is remote. The person, the room and personal effects are the chief sources of the contagion. The fact that small-pox has been stamped out in all civilized communities is another remarkable instance of what preventive means can do. The most efficient means of checking the progress of contagion to by isolation and disinfection, but the greatest of all is quarantine when cholera is suspected. If it be not allowed to spread from house to house the city is safe. If the fire be not allowed to spread beyond the burning building the city is saved.

The physician should be a sanitarian, because he knows the ways and means by which many diseases can be averted. He need not fear criticism. Families to whom he is called will gladly listen to such information. The physician will instruct the family to isolate the sick child who has a contagious disease from other children—to have the sick room as remote as possible—to have no communication between the infected child and the well children—to have all clothing fumigated or destroyed which has been used in the room—the room thoroughly disinfected. We well know that diseases become epidemic from lack of perfect isolation, from *tack* neglect of this in the first stage, neglect in allowing well children to come in contact with the sick too early in their convalescence. While yet there is a discharge from the nose or throat in scarlatina or diphtheria, or before the desquamative process is completed. The physician should give the time when for its own safety it can go upon the street or public places. He should impress upon the minds of parents and nurses that too early meeting with well children is a criminal offense against the health of the community. Preventive measures can never avail to accomplish the great purpose of lessening disease until sanitary rules are placed upon the statutes backed by intelligent public opinion. The people have been educated to the idea of the cure instead of the avoidance of disease. The doctors should popularize sanitation.

This easily becomes a part of his life work as a professional life saver. The intelligent mothers will be his best co-workers. They will adopt quickly all rules prescribed with reference to dress, ventilation, hours of study and play, every thing conducive to the health of the children. The press will be willing to devote a little space to the sanitary profession for the spread of such knowledge. People are more apt to believe what they read in sanitary publications and papers than what they hear spoken.

In this way they will have hygienic information served to them from a source which will be often before them. Teachers will thus become more mindful of the ventilation of their school rooms and act in concert with the local boards of health.

They will not allow a child to enter the school enclosure sent from an infected house.

Public opinion will count it reprehensible for mothers to send such child upon the street to play with other children until the danger is over.

Public opinion will not allow a child ailing with contagious disease to enter a crowded car or audience room, obliging a mother to flee from there with her well child. The time is coming when the contagious diseases of childhood will not be looked upon as inevitable. There was a time, and now is, among the less informed people, when the children were thought to be destined to have all the diseases from the itch to scarlatina.

A mother has been known to send her well child to a neighbor's house to "catch" measles or whooping-cough. Another ignorant mother of fatalistic delusion once said to me, "If my child is to have diphtheria it will have it no matter how great my caution. If my child is not to have the disease it will not contract it even if it play face to face with a child having the disease in its worst form."

I once visited a child who had diphtheria—a neighbor woman came with her little child to see the sick one. The children were together almost mouth to mouth. I asked the brutal mother if she loved her child. She said yes; then take your child out of this room, I said—telling her of the danger. She said her child was as safe there as any where else. In a few days the little innocent visitor died of the disease. The remote cause of its death was criminal exposure by its natural protector.

A parent having this barbarian error of belief is a source of danger to the family and the neighborhood.

The physician who hears such opinions expressed has an opportunity of doing the public a service as missionary. I have been witness of the neglect of isolating children notwithstanding our advice to limit the disease. It would by contact be carried from tenement to tenement, passed around from child to child as if it were something every family with children ought to have.

Diseases are avoided by understanding how they are induced.

Pneumonia is avoided during the cold months by having rooms of as low temperature as is compatible with comfort and adequate woolen clothing. We cannot control the temperature without—the artificial weather within we can control. If we subject ourselves to high temperature and humidity indoors, then to that of winter cold with insufficient dress we have exposed ourselves to the danger of contracting pneumonia and other diseases of the respiratory organs.

We avoid phthisis by removing to a warm and equitable climate where it does not prevail.

The cholera infantum by removing the child from the heated city to the seashore or to the cool mountain air.

We hail the age where it can be oftener said,

"Of no distemper, of no blast he died,
But fell like autumn fruit that mellowed long;
Even wondered at, because he dropt no sooner.
Fate seemed to wind him up for fourscore years;
Yet freshly ran he on ten winters more;
Till like a clock worn out with eating time,
The wheels of weary life at last stood still."

Stafford Springs, Conn. C. B. Newton, M. D.

